# Anthropometric measures at different ages and endometrial cancer risk

**DOI:** 10.1038/bjc.2011.63

**Published:** 2011-03-08

**Authors:** L Dal Maso, A Tavani, A Zucchetto, M Montella, M Ferraroni, E Negri, J Polesel, A Decarli, R Talamini, C La Vecchia, S Franceschi

**Affiliations:** 1 SOC Epidemiologia e Biostatistica, Centro di Riferimento Oncologico, 33081 Aviano (PN), Italy; 2 Dipartimento di Medicina del Lavoro, Sezione Statistica Medica e Biometria ‘GA Maccacaro’, Università degli Studi di Milano, 20133 Milano, Italy; 3 Dipartimento di Epidemiologia, Istituto di Ricerche Farmacologiche ‘Mario Negri’, 20156 Milano, Italy; 4 Servizio di Epidemiologia, Istituto Tumori ‘Fondazione Pascale’, 80131 Napoli, Italy; 5 International Agency for Research on Cancer, 69372 Lyon, France

**Keywords:** endometrial cancer, body mass index, waist-to-hip ratio, case–control studies

## Abstract

**Background:**

Endometrial cancer is strongly associated with body mass index (BMI), but the influence of BMI history and of different types of obesity is uncertain.

**Ethods:**

M A case–control study was carried out in Italy including 454 cases and 908 controls admitted to hospital for acute non-hormone-related conditions. Odds ratios (ORs) and 95% confidence intervals (CIs) were computed using multivariate logistic and spline regression models.

**Results:**

The OR for BMI >30 at diagnosis compared with 20 to <25 kg m^−2^ was 4.08 (95% CI: 2.90–5.74). The association for BMI was monotonic with a possible steeper increase for BMI above 28. Conversely, waist-to-hip ratio (WHR) showed a bell shaped curve with increased OR (2.10; 95% CI: 1.43–3.09) in the intermediate tertile only. After stratification by BMI at diagnosis, history of weight loss and BMI at age 30 did not influence endometrial cancer risk. History of obesity in middle age had a weak and not significant adverse effect among obese women (OR=1.60; 95% CI: 0.52–4.96).

**Conclusion:**

The predominant importance of recent weight compared to lifetime history, justifies encouraging weight reduction in women at any age.

Endometrial cancer is strongly associated with overweight and obesity (Levi *et al*, 1992; La Vecchia *et al*, 1997; Calle *et al*, 2003), which account for 30% (∼16 000 new cases per year) of cases among European women (Renehan *et al*, 2010). This association reflects the relationship between adiposity and high levels of unopposed oestrogens, which derive from increased frequency of anovulatory cycles (in pre-menopausal women) or the enhanced conversion of adrenal androgens into oestrogens in adipose tissue (in post-menopausal women; Key and Pike, 1988; Parazzini *et al*, 1991). The excess risk for endometrial cancer increases with age, reflecting the increasing importance of androgen conversion as ovarian activity has ceased (Key and Pike, 1988; Parazzini *et al*, 1991).

Some studies have suggested that, among women of normal body mass index (BMI) at diagnosis, there is little additional excess risk of endometrial cancer in relation to history of overweight (Le Marchand *et al*, 1991; Levi *et al*, 1992; Shu *et al*, 1992; Swanson *et al*, 1993; Olson *et al*, 1995; Terry *et al*, 1999; Weiderpass *et al*, 2000; Schouten *et al*, 2004; Xu *et al*, 2005; Trentham-Dietz *et al*, 2006; Chang *et al*, 2007; Friedenreich *et al*, 2007; WCRF/AICR – World Cancer Research Fund/American Institute for Cancer Research, 2007; Park *et al*, 2010). However, data on the relationship between lifetime changes in BMI and endometrial cancer risk are limited and difficult to assess because a monotonic pattern (gradual weight increases during life) predominates in most studied populations (Le Marchand *et al*, 1991; Levi *et al*, 1992; Swanson *et al*, 1993; Olson *et al*, 1995; Xu *et al*, 2005, 2006; Park *et al*, 2010). Similarly, there is limited information on the influence on endometrial cancer risk of different types of obesity, in particular, waist-to-hip ratio (WHR) (WCRF/AICR – World Cancer Research Fund/American Institute for Cancer Research, 2007). In some studies from Europe (Friedenreich *et al*, 2007), North America (Austin *et al*, 1991; Schapira *et al*, 1991; Swanson *et al*, 1993; Goodman *et al*, 1997), and China (Shu *et al*, 1992; Xu *et al*, 2005), BMI and waist circumference were stronger predictors of endometrial cancer risk than WHR, but the issue remains open to discussion.

To further explore these issues, we used data on BMI at different ages and measures of waist and hip at diagnosis from a case–control study on endometrial cancer carried out in different parts of Italy (Zucchetto *et al*, 2009).

## Materials and methods

A case–control study on endometrial cancer was conducted between 1992 and 2006 in three Italian areas: Pordenone and Milan in the north and Naples in the south (Lucenteforte *et al*, 2008; Zucchetto *et al*, 2009). Cases were 454 women (median age 60 years, range 18–79 years) with histologically confirmed endometrial cancer and no previous history of cancer; those diagnosed less than 1 year before recruitment were eligible. Controls were 908 women (median age 61 years, range 19–79 years) admitted to the same network of hospitals of cases for a wide spectrum of non-neoplastic, acute illnesses. Women with a history of hysterectomy or admitted for gynaecological or hormone-related conditions were not eligible as controls. The reasons for hospital admission among controls were trauma (36%), orthopaedic diseases (32%), acute surgical conditions (9%), and other illnesses (eye, nose, ear, skin, or dental disorders, 23%). Cases and controls were frequency matched on study centre and 5-year age, with a 1 : 2 ratio.

Centrally trained staff interviewed eligible women during their hospital stay. Less than 5% of the approached cases and controls refused the interview. The response rates were similar across hospitals and geographic areas. All interviews were conducted using a structured questionnaire, which included information on age, education and other socioeconomic factors, physical activity, smoking habit, alcohol intake, a validated food frequency questionnaire, a problem-oriented medical history, and history of cancer in first degree relatives. In a detailed section of the questionnaire, women were asked to report their height and weight at 1 year before cancer diagnosis or interview (for controls; referred to, for brevity, as measures at diagnosis), weight at age 30 and 50 years, lifelong highest and lowest weight, and perceived body size at age 12 years (i.e., thinner than, same as, heavier than peers). BMI was computed as weight divided by squared height (kg m^−2^). The interviewers measured the circumference of the waist (2 cm above the umbilicus) and hip (maximal protrusion) at the time of interview and computed WHR. Waist-to-height ratio (WHtR) was also computed. Waist or hip could not be measured in 89% of women interviewed in Milan centre, leading to a lack of information for WHR and WHtR in 33% of cases and 35% of controls. However, in the overall study, median BMI in women with a measured WHR was not substantially different from that in women for whom the information on WHR or WHtR was missing (26.4 and 26.2, respectively, among cases and 26.1 and 25.8 among controls).

Standard BMI categories (<20, 20 to <25, 25 to <30, and ⩾30 kg m^−2^) were used to facilitate comparisons with previous studies. Tertiles obtained from the combined distribution of cases and controls were used to assess other anthropometric measures. A conditional logistic regression model was used to compute odds ratios (ORs) and the corresponding 95% confidence intervals (CIs). All analyses were conditioned on age and study centre and adjusted for calendar period of interview, years of education, smoking habits, age at menarche and at menopause, parity, and use of oral contraceptives and hormone replacement therapy. Additional adjustment for alcohol intake, and occupational and recreational physical activity did not materially modify the risk estimates. To avoid potentially arbitrary categorisations, the ‘dose–risk’ relationship between BMI at diagnosis or WHR and endometrial cancer risk was assessed using logistic cubic regression splines (Greenland, 1995; Rosenberg *et al*, 2003; Dal Maso *et al*, 2007), and appropriate point-wise CIs were computed. The optimal number of segments of BMI or WHR was selected in order to minimise the Akaike information criterion (Akaike, 1973).

## Results

Table 1 shows the distribution of 454 endometrial cancer cases and 908 controls according to matching variables (age and study centre) and potential confounders. By design, cases and controls had equal distribution of age and study centre. No association was found with education or smoking status. Endometrial cancer risk was inversely associated with age at menarche, parity, and oral contraceptive use, whereas directly associated with age at menopause (Zucchetto *et al* 2009).

The distribution of endometrial cancer cases and controls, and the corresponding ORs, according to height, weight, BMI at diagnosis and at different ages are shown in Table 2. An inverse association with height was observed (OR=0.71; 95% CI: 0.53–0.95; for women ⩾165 cm tall compared with <160 cm). Weight and BMI were directly related to endometrial cancer risk; compared with normal weight women (BMI 20 to <25 kg m^−2^), the ORs were 0.56 (95% CI: 0.27–1.15) in women with BMI <20, 1.41 (95% CI: 1.05–1.90) and 4.08 (95% CI: 2.90–5.74) in women with BMI 25 to <30 and BMI ⩾30, respectively. OR was 1.45 (95% CI: 1.06–1.98) among women who reported to have been heavier than their peer group at age 12 years. Compared with BMI 20 to <25, the OR for BMI ⩾30 at age 30 was 1.78 (95% CI: 1.01–3.14), and 3.37 (95% CI: 2.26–5.04) for BMI⩾30 at age 50 (Table 2). Decreases from highest BMI by ⩾2 kg m^−2^ were weakly associated with reductions in endometrial cancer risk (OR *vs* no decrease in BMI during lifetime=0.80; 95% CI: 0.59–1.08), but the association disappeared by adjustment for BMI at diagnosis (OR *vs* no decrease in BMI during lifetime=0.96; 95% CI: 0.70–1.32; data not shown).

Table 3 shows the relationship between waist and hip circumferences, WHR, and WHtR and endometrial cancer risk. Significant trends of risk emerged, with the increase of waist circumference (OR for ⩾96 *vs* <84 cm: 2.68; 95% CI: 1.78–4.03), hip circumference (OR for ⩾109 *vs* <100 cm=2.49; 95% CI: 1.66–3.72), and WHtR (OR for ⩾0.59 *vs* <0.52=3.10; 95% CI: 2.03–4.73). No linear trend in risk, however, was observed for WHR. Compared with the lowest WHR tertile (<0.833), the OR was 2.10 (95% CI: 1.43–3.09) in the intermediate tertile but 1.33 (95% CI: 0.89–1.97) in the highest tertile (⩾0.890).

Table 4 shows the relationship between height, WHR and WHtR, and endometrial cancer risk in strata of BMI at diagnosis. The tendency of height to be inversely associated with risk was restricted to overweight and obese women. An increased risk only in intermediate tertile of WHR was confirmed within all three BMI strata. Conversely, a nonsignificant direct association with WHtR was exclusively observed among normal weight women.

**Figure 1:**
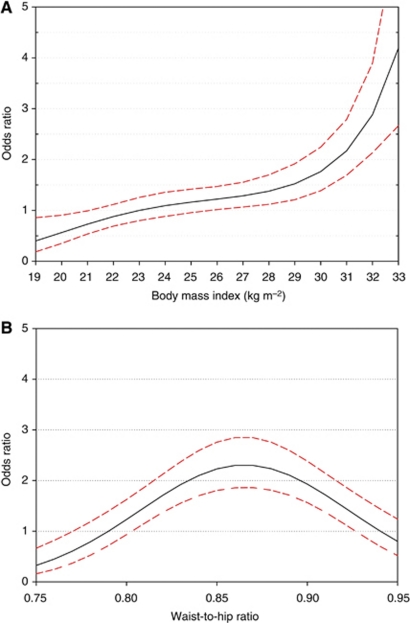
Estimates of odds ratios and 95% confidence intervals of endometrial cancer by body mass index at diagnosis (**A**) and waist-to-hip ratio (**B**), using cubic regression splines. Italy, 1992–2006 (Odds ratios from regression equations include terms for age, study centre, year of interview, education, smoking status, age at menarche, age at menopause, oral contraceptives use, parity, and hormone replacement therapy use. Curves are shown for best-fitting cubic spline regression models according to Akaike Information Criterion. Dashed lines represent 95% confidence intervals. Ranges represent the distribution of variables among controls from 10th to 90th percentile). Reference categories were body mass index=23 and waist-to-hip ratio=0.79.

The shapes of the best-fitting regression splines for BMI and WHR are shown in Figure 1. Reference level was set to the median values of BMI (23) and WHR (0.79) of the reference categories in the Tables 2 and 3. The association of endometrial cancer risk with BMI did not show a lower threshold, was monotonic, and risk increase was steeper after a BMI of ∼28 (Figure 1A). Conversely, the relationship between risk and WHR was bell shaped and the corresponding OR was greatest for WHR between 0.86 and 0.87 (Figure 1B). When the associations of endometrial cancer risk with BMI and WHR were examined within strata of women with different characteristics, no statistically significant heterogeneity was observed by education, smoking habit, and occupational or recreational physical activity. However, the association with BMI was somewhat stronger among post-menopausal women (OR for BMI ⩾30 *vs* 20 to <25=4.94; 95% CI: 3.38–7.23) than among pre- and peri-menopausal women (OR=2.12; 95% CI: 0.92–4.91), though this difference was not statistically significant (*χ*^2^ for heterogeneity=2.04; *P*=0.36).

Table 5 shows the association of endometrial cancer risk with BMI at ages 30 and 50 years within strata of women who had similar BMI at diagnosis. Some categories of BMI at age 30 and 50 years had to be combined on account of the small numbers reporting large BMI variations. Among women with BMI <25 at diagnosis, the OR for BMI ⩾25 *vs* <25 was 1.24 (95% CI: 0.49–3.13) and 1.59 (95% CI: 0.71–3.52) at ages 30 and 50 years, respectively. Among women with BMI ⩾30 at diagnosis, the ORs were 1.23 (95% CI: 0.54–2.82) and 1.60 (95% CI: 0.52–4.96) for a BMI ⩾30 *vs* <25 at ages 30 and 50 years, respectively. No association of perceived body size during adolescence and endometrial cancer emerged after stratification for BMI at diagnosis (data not shown).

## Discussion

Our case–control study confirms the strong relationship between weight and BMI at diagnosis and endometrial cancer risk, especially among post-menopausal women. The effect of BMI did not show a lower threshold: but the risk curve became steeper among severely overweight women (BMI >28). After adjustment or stratification by BMI at diagnosis, history of weight loss and BMI in young adulthood did not influence endometrial cancer risk. History of obesity in middle age had, however, a weak nonsignificant adverse effect among obese women aged 50 years or older. These findings provide indirect support to the possibility of weight excess acting as late-stage carcinogens (Parazzini *et al* 1991; La Vecchia *et al* 1997). Our BMI results are in broad agreement with previous work and with a meta-analysis that showed summary risk estimates of 1.52 (95% CI: 1.35–1.72) in 15 cohort studies and 1.56 (95% CI: 1.45–1.66) in 28 case–control studies for an increase of 5 BMI units (WCRF/AICR – World Cancer Research Fund/American Institute for Cancer Research, 2007). The association of BMI with endometrial cancer risk in our study showed no lower threshold and was nonlinear, in agreement with the findings of two meta-analyses (Crosbie *et al*, 2010; Renehan *et al*, 2010) that found a highly marked increase in risk for a BMI above 27.

The relationship between endometrial cancer risk and WHR is less clear. A meta-analysis of one cohort study and four case–control studies provided a summary risk estimate of 1.45 (95% CI: 1.00–2.09) for an increase of 0.1 WHR units (WCRF/AICR – World Cancer Research Fund/American Institute for Cancer Research, 2007). In the Iowa Women's Health cohort study (Folsom *et al*, 2000) and in a case–control study (Goodman *et al*, 1997), included in the meta-analysis (WCRF/AICR – World Cancer Research Fund/American Institute for Cancer Research, 2007), the association with WHR disappeared after allowance for BMI. Out of three subsequent cohort studies, the European Prospective Investigation into Cancer and nutrition (EPIC; Friedenreich *et al*, 2007), the Women's Health Study (Conroy *et al*, 2009), and the California Teachers Study (Canchola *et al*, 2010), two showed a direct association with WHR (Friedenreich *et al*, 2007; Canchola *et al*, 2010), but in both studies the upper quantiles of WHR (>0.83 and ⩾0.80, respectively) were lower than in this study. The reasons for a bell-shaped relationship between endometrial cancer risk and WHR are not clear, but may be related to the hormonal correlates of different types of fat distribution. The effect of body fat distribution seemed to be weaker and more complex than the effect of weight excess; risk was clearly associated with both waist and hip circumference, but the relationship with WHR had a bell shape, at least among postmenopausal women (80% of study women).

WHtR is another measure of abdominal adiposity that has been only rarely used in endometrial cancer studies (Canchola *et al*, 2010). WHtR is considered a measure of visceral fat independent of height. The overall direct association with WHtR in this study was stronger than the association with WHR but seemed to be restricted to normal weight women.

The key interpretation of the relationship between overweight, obesity, and endometrial cancer is in terms of the ‘unopposed oestrogen’ hypothesis (Key and Pike, 1988, Parazzini *et al*, 1991), that is, of relative excess of oestrogens, following anovulation in pre-menopause, and androgen conversion to oestrogens in the adipose tissue in post menopause. In the EPIC study, endometrial cancer risk in post-menopausal women was directly associated with the levels of oestrogens and with testosterone, but not with the levels of androstenedione and dehydroepiandrosterone (Allen *et al*, 2008). These findings may explain the weaker association with WHR than for BMI, in our and other studies (Austin *et al*, 1991; Shu *et al*, 1992; Goodman *et al*, 1997; Folsom *et al*, 2000; Friedenreich *et al*, 2007; Conroy *et al*, 2009). WHR is chiefly a marker of androgenic obesity and hence not strongly related to oestrogen excess (Seidell *et al*, 1990, 1989). In one study, WHR was associated to breast cancer risk in oestrogen receptor negative, but not in oestrogen receptor positive tumours (Harris *et al*, 2011).

In agreement with previous work (WCRF/AICR – World Cancer Research Fund/American Institute for Cancer Research, 2007; Crosbie *et al*, 2010), the association of endometrial cancer risk with BMI was stronger in post-menopausal women than in pre- and peri-menopausal women on account of the stronger importance of oestrogens deriving from androgen conversion after ovarian activity had ceased. No risk correlates other than menopausal status modified the association with BMI in our study.

Only a few studies have considered the relationship with lifetime history of body weight and endometrial cancer risk. A meta-analysis of BMI in young adulthood showed summary estimates of 1.31 (95% CI: 1.12–1.54) per 5 BMI unit increase, based on three cohort studies. The corresponding value, based on six case–control studies, was 1.10 (95% CI: 0.95–1.27; WCRF/AICR – World Cancer Research Fund/American Institute for Cancer Research, 2007). Four prospective studies showed a significant direct association between weight gain during life (measured as weight or BMI variation; Schouten *et al*, 2004; Chang *et al*, 2007; Friedenreich *et al*, 2007; Park *et al*, 2010), whereas another showed no association (Terry *et al*, 1999). Five case–control studies also reported a direct association of weight gain with endometrial cancer risk (Levi *et al*, 1992; Swanson *et al*, 1993; Olson *et al*, 1995; Xu *et al*, 2005; Trentham-Dietz *et al*, 2006), whereas another study found no relationship (Weiderpass *et al*, 2000). A case–control study from China showed a direct relation with weight at ⩾50 years, but not at younger age (Shu *et al*, 1992). In our study, we made a special effort to separate the effects of BMI in young adulthood and middle age and of lifetime weight changes from the effect of BMI at cancer diagnosis or interview (controls). We found no evidence of an influence of history of overweight and obesity after stratification by BMI at diagnosis. Our findings are, therefore, consistent with excess weight affecting only late carcinogenesis stages. Only obesity at age 50 years showed an association of borderline statistical significance among women older with BMI ⩾30 at diagnosis. This finding is compatible with an effect of duration of exposure to high levels of circulating oestrogen levels after menopause.

No association or a weak direct association has been reported between height and endometrial cancer risk (WCRF/AICR – World Cancer Research Fund/American Institute for Cancer Research, 2007; Friedenreich *et al*, 2007; Park *et al*, 2010). Conversely, we found a weak inverse association, in agreement with some previous Italian (La Vecchia *et al*, 1997) and Swiss studies (Levi *et al*, 1992). However, in our study the association with height was restricted to overweight and obese women and was accompanied by a tendency of control women having BMI⩾25 to be shorter than leaner women (data not shown).

Case–control studies may be subject to selection and information bias (Breslow and Day, 1980). However, possible sources of selection bias are limited in our study, as cases and controls were drawn from the same catchment areas, participation was almost complete, and women with diseases potentially related to diet and dietary modifications were excluded from the control group. The high proportion of missing values for WHR cannot be a source of bias as it derives from lack of measurement of waist or hip in the vast majority of women from one centre.

In our study, weights during lifetime and height were self-reported. It is known that most individuals, and especially those overweight, tend to underestimate their weight (WCRF/AICR – World Cancer Research Fund/American Institute for Cancer Research, 2007). Conversely, height tends to be systematically overestimated (WCRF/AICR – World Cancer Research Fund/American Institute for Cancer Research, 2007). As for lifetime weight history, past body measures were generally well correlated with corresponding measures (Casey *et al* 1991), even in older people (Klipstein-Grobusch *et al*, 1998). A differential recall between cases and control is possible, but we do not think that weight and height were differentially reported by cases and controls. All women in our study were interviewed in similar hospital settings, and the general population was unaware of the possible link between anthropometric measures and endometrial cancer. In addition, opposite to many other cancers, endometrial cancer is not frequently preceded by weight loss (De Vita *et al*, 2007).

Confounding was dealt with in our study by adjusting for a broad range of risk correlates, including reproductive and hormone-related factors. Less than 6% of women reported any use of lifetime hormone replacement therapy for 2 or more years (Zucchetto *et al*, 2009), thus avoiding the need to exclude users from the assessment of the effects of anthropometric measures.

The implications of this report from a public health viewpoint are clear. The fraction of endometrial cancer cases attributable to overweight and obesity in our study population was of 41% (95% CI: 31–51% Mezzetti *et al*, 1996), even higher than the estimated 30% (95% CI: 26–34%) reported for the combination of 30 European countries (Renehan *et al*, 2010). The predominant importance of recent weight, compared to lifetime history, justifies encouraging weight reduction in women at any age.

## Figures and Tables

**Table 1:** Distribution of 454 endometrial cancer cases and 908 controls according to selected variables, Italy, 1992–2006

	**Cases**		**Controls**	
	**No.**	**%**	**No.**	**%**
*Age (years)*				
<50	67	14.8	134	14.8
50–59	140	30.8	280	30.8
60–69	166	36.6	332	36.6
⩾70	81	17.8	162	17.8
				
*Study center*				
Aviano–Pordenone	237	52.2	474	52.2
Milan	140	30.8	280	30.8
Naples	77	17.0	154	17.0
				
*Education (years)*				
<7	263	57.9	553	60.9
7–11	119	26.2	225	24.8
⩾12	72	15.9	130	14.3
				
*Smoking status*				
Never	331	72.9	647	71.3
Current	75	16.5	157	17.3
Former	48	10.6	104	11.5
				
*Age at menarche (years)* a				
<11	24	5.3	44	4.9
11–13	283	62.6	475	52.7
14–16	137	30.3	345	38.3
⩾17	8	1.8	38	4.2
				
*Parity*				
Nulliparous	68	15.0	126	13.9
Parous	386	85.0	782	86.1
				
*Oral contraceptive use*				
Never	408	89.9	790	87.0
Ever	46	10.1	118	13.0
				
*Hormone replacement therapy*				
Never	405	89.2	830	91.4
Ever	49	10.8	78	8.6
				
*Menopausal status* a				
Pre/peri	85	19.2	174	19.3
Post	358	80.8	726	80.7

a The sum does not add up to the total because of some missing values.

**Table 2:** Distribution of 454 endometrial cancer cases and 908 controls, and corresponding odds ratio (OR) with 95% confidence intervals (CIs)^a^, according to body mass index (BMI) at diagnosis and at different ages,^b^ Italy, 1992–2006

	**Cases**		**Controls**		
	**No.**	**(%)**	**No.**	**(%)**	**OR (95% CI)**
*Height (cm)*					
<160	152	(33.5)	258	(28.5)	1^c^
160–164	148	(32.6)	280	(31.0)	0.90 (0.66–1.21)
⩾165	154	(33.9)	366	(40.5)	0.71 (0.53–0.95)
*χ*^2^ for trend (*P*-value)					5.39 (*P*=0.02)
					
*Weight (kg)*					
<64	109	(24.0)	355	(39.1)	1^c^
64–74	145	(31.9)	311	(34.3)	1.51 (1.10–2.06)
⩾75	200	(44.1)	242	(26.7)	2.71 (1.99–3.70)
*χ*^2^ for trend (*P*-value)					40.17 (*P*<0.01)
					
*Body mass index (kg m* ^*−2*^ *)*					
<20	11	(2.4)	58	(6.4)	0.56 (0.27–1.15)
20 to <25	115	(25.3)	355	(39.3)	1^c^
25 to <30	160	(35.2)	351	(38.8)	1.41 (1.05–1.90)
⩾30	168	(37.0)	140	(15.5)	4.08 (2.90–5.74)
*χ*^2^ for trend (*P*-value)					67.95 (*P*<0.01)
					
BMI (kg m^−2^) 5-Unit increase					1.89 (1.65–2.17)
					
*Perceived body size at age 12 years*					
Thinner than peers	146	(32.3)	351	(39.1)	1^c^
Same than peers	173	(38.3)	341	(38.0)	1.12 (0.85–1. 94)
Heavier than peers	133	(29.4)	206	(22.9)	1.45 (1.06–1.98)
*χ*^2^ trend (*P*-value)					5.19 (*P*=0.02)
					
*BMI at age 30 years*^d^ *(kg m*^*−2*^*)*					
<20	55	(12.6)	179	(21.9)	0.57 (0.40–0.83)
20 to <25	252	(57.8)	473	(57.8)	1^c^
25 to <30	100	(22.9)	134	(16.4)	1.40 (1.02–1.95)
⩾30	29	(6.7)	33	(4.0)	1.78 (1.01–3.14)
*χ*^2^ for trend (*P*-value)					18.95 (*P*<0.01)
					
*BMI at age 50 years*^e^ *(kg m*^*−2*^*)*					
<20	7	(1.9)	54	(7.7)	0.39 (0.17–0.91)
20 to <25	138	(37.3)	339	(48.3)	1^c^
25 to <30	129	(34.9)	223	(31.9)	1.48 (1.08–2.04)
⩾30	96	(26.0)	84	(12.0)	3.37 (2.26–5.04)
*χ*^2^ for trend (*P*-value)					42.33 (*P*<0.01)

a ORs from conditional logistic regression models, conditioned on age and study centre, adjusted for year of interview, education, smoking status, age at menarche, age at menopause, oral contraceptive use, parity, and hormone replacement therapy use.

b The sum does not add up to the total because of some missing values.

c Reference category.

d Women ⩾30 years old only.

e Women ⩾50 years old only.

**Table 3:** Distribution of 454 endometrial cancer cases and 908 controls, and corresponding odds ratio (OR) with 95% confidence intervals (CIs),^a^ according to measures of fat distribution,^b^ Italy, 1992–2006

	**Cases**		**Controls**		
	**No.**	**(%)**	**No.**	**No.**	**OR (95% CI)**
*Waist circumference (cm)*					
<84	79	(25.7)	221	(37.2)	1^c^
84–95	101	(32.9)	226	(38.1)	1.22 (0.83–1.79)
⩾96	127	(41.4)	147	(24.8)	2.68 (1. 78–4.03)
*χ*^2^ for trend (*P*-value)					22.51 (*P*<0.01)
					
*Hip circumference (cm)*					
<100	87	(28.4)	218	(36.8)	1^c^
100 to108	96	(31.4)	204	(34.5)	1.35 (0.92–1.98)
⩾109	123	(40.2)	170	(28.7)	2.49 (1.66–3.72)
*χ*^2^ for trend (*P*-value)					18.99 (*P*<0.01)
					
*Waist-to-hip ratio*					
<0.833	71	(23.3)	224	(37.8)	1^c^
0.833 to <0.890	129	(42.2)	177	(29.9)	2.10 (1.43–3.09)
⩾0.890	106	(34.6)	191	(32.3)	1.33 (0.89–1.97)
*χ*^2^ for trend (*P*-value)					1.38 (*P*=0.24)
					
*Waist-to-height ratio*					
<0.52	77	(25.1)	237	(40.0)	1^c^
0.52 to <0.59	101	(32.9)	200	(33.8)	1.66 (1.12–2.46)
⩾0.59	129	(42.0)	155	(26.1)	3.10 (2.03–4.73)
*χ*^2^ for trend (*P*-value)					27.53 (*P*<0.01)

a ORs from conditional logistic regression models, conditioned on age and study centre, adjusted for year of interview, education, smoking status, age at menarche, age at menopause, oral contraceptives use, parity, and hormone replacement therapy use.

b The sum does not add up to the total because of some missing values.

c Reference category.

**Table 4:** Odds ratio (OR) with 95% confidence intervals (CIs)^a^ of 454 endometrial cancer cases (CA) and 908 controls (CO), according to height, waist-to-hip ratio, and waist-to-height ratio in strata of recent body mass index at diagnosis, Italy, 1992–2006

	**Body mass index (kg m^−2^) at diagnosis**					
	**<25**		**25 to <30**		**⩾30**	
	**CA : CO**	**OR (95% CI)**	**CA : CO**	**OR (95% CI)**	**CA : CO**	**OR (95% CI)**
*Height (cm)*						
<160	29 : 103	1^b^	52 : 102	1^b^	71 : 53	1^b^
160–164	42 : 126	1.25 (0.69–2.25)	53 : 110	0.93 (0.56–1.54)	53 : 44	0.97 (0.53–1.79)
⩾165	55 : 184	1.13 (0.65–1.96)	55 : 139	0.74 (0.44–1.23)	44 : 43	0.69 (0.37–1.30)
						
*Waist-to-hip ratio*						
<0.833	33 : 135	1^b^	22 : 70	1^b^	16 : 19	1^b^
0.833 to <0.890	29 : 71	1.70 (0.87–3.34)	50 : 78	1.74 (0.88–3.45)	50 : 28	2.95 (1.04–8.39)
⩾0.890	17 : 60	1.01 (0.49–2.09)	38 : 88	1.11 (0.55–2.25)	51 : 43	1.17 (0.43–3.15)
						
*Waist-to-height ratio*						
<0.52	55 : 195	1^b^	22 : 41	1^b^	0 : 1	0 (—)
0.52 to <0.59	21 : 64	1.68 (0.84–3.36)	59 : 122	0.92 (0.45–1.87)	21 : 14	1^b^
⩾0.59	4 : 6	4.69 (0.97–22.76)	29 : 74	0.82 (0.35–1.90)	96 : 75	0.80 (0.31–2.05)

a ORs from conditional logistic regression models, conditioned on age and study centre, adjusted for year of interview, education, smoking status, age at menarche, age at menopause, oral contraceptives use, parity, and hormone replacement therapy use.

b Reference category.

**Table 5:** Distribution of 454 endometrial cancer cases and 908 controls, and corresponding odds ratio (OR) with 95% confidence intervals (CIs)^a^, by body mass index (BMI) at ages 30 and 50 years in strata of BMI at diagnosis,^b^ Italy, 1992–2006

	**BMI at age 30 years^c^**					**BMI at age 50 years^d^**				
	**Cases**		**Controls**			**Cases**		**Controls**		
**BMI (kg m^−2^) at diagnosis**	**No.**	**(%)**	**No.**	**(%)**	**OR (95% CI)**	**No.**	**(%)**	**No.**	**(%)**	**OR (95% CI)**
*<25*										
<25	114	(93.4)	351	(93.4)	1^e^	79	(85.9)	270	(89.4)	1^e^
⩾25	8	(6.6)	25	(6.7)	1.24 (0.49–3.13)	13	(14.1)	32	(10.6)	1.59 (0.71–3.52)
										
*⩾25 to <30*										
<25	117	(76.5)	237	(75.5)	1^e^	55	(42.0)	113	(40.5)	1^e^
25 to <30	31	(20.3)	64	(20.4)	0.99 (0.58–1.68)	66	(50.4)	147	(52.7)	1.02 (0.63–1.65)
⩾30	5	(3.3)	13	(4.1)	0.95 (0.30–3.02)	10	(7.6)	19	(6.8)	1.70 (0.66–4.35)
*χ*^2^ for trend (*P*-value)					0.01 (*P*=0.93)					0.52 (*P*=0.47)
										
*⩾30*										
<25	76	(47.2)	64	(49.6)	1^e^	11	(7.5)	10	(8.4)	1^e^
25 to <30	61	(37.9)	48	(37.2)	1.12 (0.62–2.00)	51	(34.7)	48	(40.2)	0.89 (0.29–2.76)
⩾30	24	(14.9)	17	(13.2)	1.23 (0.54–2.82)	85	(57.8)	61	(51.3)	1.60 (0.52–4.96)
*χ*^2^ for trend (*P*-value)					0.29 (*P*=0.59)					

a ORs from conditional logistic regression models, conditioned on age and study centre, adjusted for year of interview, education, smoking status, age at menarche, age at menopause, oral contraceptives use, parity, and hormone replacement therapy use, when appropriate.

b The sum does not add up to the total because of some missing values.

c Women ⩾30 years old only.

d Women ⩾50 years old only.

e Reference category.
